# Cost-effectiveness analysis of segmental adrenal venous sampling with radiofrequency ablation for primary aldosteronism in Japan

**DOI:** 10.1007/s11604-024-01665-6

**Published:** 2024-09-25

**Authors:** Satoru Yanagaki, Kei Omata, Sota Oguro, Hideki Ota, Tomomi Sato, Hiroki Kamada, Hiromitsu Tannai, Yuta Tezuka, Yoshikiyo Ono, Miho Sato, Hiroyuki Ohbe, Kei Takase

**Affiliations:** 1 Department of Diagnostic Radiology, Tohoku University Graduate School of Medicine, Sendai, Miyagi Japan; 2 Department of Diagnostic Radiology, Tohoku Medical Pharmaceutical University Hospital, Sendai, Miyagi Japan; 3 Department of Diabetes, Metabolism and Endocrinology, Tohoku University Hospital, Sendai, Miyagi Japan; 4 Division of Nephrology, Rheumatology and Endocrinology, Tohoku University Graduate School of Medicine, Sendai, Miyagi Japan; 5 Division of Radiological Examinations and Technology, Tohoku University Graduate School of Medicine, Sendai, Miyagi Japan; 6 Department of Emergency and Critical Care Medicine, Tohoku University Hospital, Sendai, Miyagi Japan

**Keywords:** Primary aldosteronism, Adrenal venous sampling, Radiofrequency ablation, Cost-effectiveness analysis

## Abstract

**Purpose:**

The purpose of this study was to evaluate the cost-effectiveness of comprehensive treatment strategy, including segmental adrenal venous sampling (sAVS) and radiofrequency ablation (RFA), versus medication-only strategy for primary aldosteronism.

**Materials and methods:**

A Markov decision model was developed to compare the cost-effectiveness of a comprehensive treatment strategy and a medication-only strategy for 50-year-old men and women with stage I–III hypertension. The comprehensive treatment strategy included aldosterone/renin ratio measurement, two loading tests, computed tomography, sAVS, drugs, surgery, and RFA. We built a model with a yearly cycle over 32- and 38-year time horizons for men and women, respectively, and four health states: hypertension, heart failure, stroke, and death. The incremental cost-effectiveness ratio (ICER), expressed as Japanese yen per quality-adjusted life-years (QALYs), was estimated, and strategy preference was determined on the basis of 5 million Japanese yen per QALY societal willingness-to-pay threshold.

**Results:**

The ICERs of the comprehensive treatment strategy over the medication-only strategy were 201,482 and 3,399 JPY per QALY for men and women, respectively. The resultant ICER was less than the 5 million JPY societal willingness-to-pay threshold. Deterministic sensitivity analysis and probabilistic sensitivity analysis revealed that the results varied with the input values, but the comprehensive strategy was likely to be more cost-effective than the medication-only strategy.

**Conclusion:**

This cost-effectiveness study revealed that a comprehensive treatment strategy including sAVS and RFA was favorable compared with the medication-only strategy for managing stage I–III hypertension in 50-year-old men and women, with acceptable willingness-to-pay thresholds.

**Secondary abstract:**

This cost-effectiveness study revealed that a comprehensive treatment strategy for primary aldosteronism that included segmental adrenal sampling and radiofrequency ablation was favorable compared with the medication-only strategy for managing stage I–III hypertension in 50-year-old men and women, with acceptable willingness-to-pay thresholds.

## Introduction

Hypertension is a major risk factor for stroke and cardiovascular disease, and Japan has 40 million patients with hypertension [1–3]. In 2020, medical expenses for cardiovascular diseases, including hypertension, accounted for 19.5% of the national medical expenses for general medical care [1]. Primary aldosteronism (PA) is a major cause of secondary hypertension, and its estimated incidence accounts for 3%–20% of the cases of hypertension [1, 4, 5]. Therefore, there is a social demand for a comprehensive treatment strategy of PA that is medically and economically effective.

PA is characterized by inappropriately elevated plasma aldosterone levels, which is released from an aldosterone-producing adenoma (APA) or bilateral adrenal hyperplasia [6, 7]. In the case of APA, PA would be cured by total or partial adrenalectomy [8–11]. Therefore, it is important to localize the APA to appropriately treat hypertension caused by PA. The Japanese guidelines for PA recommend the following comprehensive treatment strategy for the diagnosis and treatment of PA: identification of an abnormal plasma aldosterone/renin ratio (ARR); at least one or two loading tests to confirm the diagnosis; computed tomography (CT); and adrenal venous sampling (AVS) to localize the adenoma [5, 12]. Therefore, the correct diagnosis and treatment of PA requires financial resources, time, and specialist staff [13].

Recent studies reported two important technical developments related to a comprehensive treatment strategy for PA. First, several studies have shown the effectiveness of segmental AVS (sAVS), which involves the collection of blood from the tributaries of the central vein to determine the intra-adrenal localization of aldosterone hypersecretion [14–17]. The authors reported that sAVS could better identify the APA in some patients than conventional AVS (cAVS), which involves the collection of blood from the bilateral adrenal central veins only. Second, some studies have reported that radiofrequency ablation (RFA) is an effective treatment for APA and may serve as a justifiable treatment alternative to surgery and medical therapy [18–21]. RFA is widely used to treat solid neoplasms, especially in patients with primary or secondary malignancies [18]. Currently, most institutions in Japan have a comprehensive treatment strategy that does not include sAVS or RFA [5]. RFA for APA has been covered by nation-wide insurance in Japan since June 2021. This is expected to lead to the further utilization of RFA for APA in Japan. Therefore, following related changes in the diagnosis and treatment of PA, the clinical significance of detecting and treating PA has become more important but more complicated than ever before.

A cost-effectiveness analysis of healthcare interventions is a method that compares different medical strategies and offers useful information for evidence-based advocacy, policy-making, and patient-care decisions [22]. The analysis is based on the costs associated with each strategy and the related effectiveness. The current reference standard for quantifying effectiveness is quality-adjusted life-years (QALYs), which are an assessment of both the quality and quantity of life [23].

Several studies have reported the cost-effectiveness of a comprehensive treatment strategy for PA [24–29]. Sato et al. reported in their cost-effectiveness analysis of diagnosing and treating PA that lifetime medical costs were higher and life-years were greater for patients with hypertension who were screened and treated via a comprehensive treatment strategy than for those treated with a medication-only strategy, with an incremental cost-effectiveness ratio (ICER) under 5 million Japanese yen (JPY) [24]. However, it is unknown whether a comprehensive treatment strategy that includes sAVS and RFA is favorable in terms of cost-effectiveness.

The purpose of this study was to evaluate the cost-effectiveness of a comprehensive treatment strategy that includes sAVS and RFA versus a medication-only strategy in patients with hypertension. In addition, the study aimed to investigate how cost-effectiveness varies across comprehensive treatment strategies with and without sAVS and RFA.

## Materials and methods

### Model structure

**Fig. 1:**
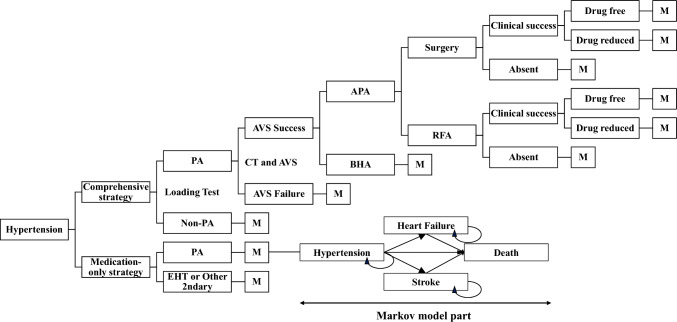
Markov decision tree model for the hypertension management of patients suspected of having primary aldosteronism. *M* Markov decision section, *PA* primary aldosteronism, *APA* aldosterone-producing adenoma, *BHA* bilateral hyperaldosteronism, *RFA* radiofrequency ablation, *CT* computed tomography, *AVS* adrenal venous sampling, *EHT* essential hypertension

We developed a combination of a decision tree model for the diagnosis and treatment phase and a Markov model for the long-term follow-up phase to evaluate the cost-effectiveness of a comprehensive treatment strategy compared with a medication-only strategy for 50-year-old men and women with stage I–III hypertension (Fig. 1). Markov processes track patients’ transitions across mutually exclusive health states that last a fixed length of time and can simulate the long-term clinical evolution of patients [30]. During each cycle, patients accumulate QALYs and costs, and transitions occur according to input probabilities. The analysis was performed from a healthcare payer perspective. We used the TreeAge Pro 2021 Healthcare module (TreeAge Software Inc., Williamstown, MA, USA) to model and simulate the cost-effectiveness. We compared five strategies in the cost-effectiveness analysis. The comprehensive treatment strategy comprised ARR measurement, two loading tests, computed tomography, sAVS, drugs, surgery, and RFA. In addition, we analyzed the following three suboptimal comprehensive treatment strategies: (i) sAVS without RFA, (ii) cAVS with RFA, and (iii) cAVS without RFA. The reference strategy was a medication-only strategy to manage hypertension using only antihypertensive drugs without ARR measurement, two loading tests, computed tomography, AVS, surgery, and RFA. Patients with PA in the medication-only strategy group were treated as patients with essential hypertension (EHT) unless the typical signs of PA or other complications developed. This model complies with both the Consolidated Health Economic Evaluation Reporting Standards 2022 (CHEERS 2022) Statement and the Japanese guidelines for cost-effectiveness analyses [31, 32].

### Decision tree model

For the diagnosis and treatment phase of PA, a decision tree model was used. The probability estimates were extracted from our hospital data and the published literature, as shown in Table 1 [14, 24, 33]. To obtain data regarding sAVS and RFA, we conducted a single-center retrospective cohort study at our hospital. Consecutive patients who were diagnosed with PA and referred to the Department of Endocrinology at Tohoku University Hospital from April 2021 to September 2022 were included. The institutional review board approved this study, and informed consent was obtained from the participants in the form of an opt-out on the website (approval number; 2021-1-979).

**Table 1:** Input data

Name	Point estimate (range)	Sources
Costs		
Costs for initial diagnosis and treatment	(Japanese yen)	
Blood test (ARR) and loading tests	17,920 (± 20%)	Our hospital data, previous research [24]
CT and AVS	570,620 (± 20%)	Our hospital data, previous research [24]
HF; initial treatment	3,463,446 (± 20%)	Our hospital data
Stroke; initial treatment	298,855 (± 20%)	Our hospital data, previous research [24]
Surgery	996,710 (± 20%)	Our hospital data, previous research [24]
RFA	677,030 (± 20%)	Our hospital data
Medication costs	(Japanese yen per year)	
Drug; EHT	60,656 (± 20%)	Our hospital data
Drug; clinical success after surgery and RFA	23,276 (± 20%)	Our hospital data
Drug; PA (APA and BHA) without surgery	61,144 (± 20%)	Our hospital data
Drug; partial success after surgery and RFA	61,144 (± 20%)	Our hospital data
HF; follow-up	80,755 (± 20%)	Our hospital data
Stroke; follow-up	191,200 (± 20%)	Our hospital data
QALY		
EHT	1.0 (± 8%)	Previous research [37]
HF	0.9 (± 8%)	Previous research [37]
Stroke	0.9 (± 8%)	Previous research [37]
Probabilities		
Prevalence of PA in HT	0.1 (± 30%)	Previous research [4, 5, 24]
Success rate of AVS	1.0 (± 30%)	Previous research [24]
Prevalence of APA (surgical PA)	0.48 (± 30%)	Our hospital data
RFA for APA	0.085 (± 30%)	Our hospital data
Clinical success rate after surgery	0.84 (± 30%)	Previous research [8, 24]
Clinical success rate after RFA	1.0 (± 30%)	Our hospital data
Drug free rate after surgery	0.44 (± 30%)	Previous research [8]
Drug free rate after RFA	1.0 (± 30%)	Our hospital data
Transition probabilities in the Markov model		
Cured PA to death	0.00416 (± 30%)	Previous research [19]
Cured PA to HF	0.0266 (± 30%)	Previous research [36]
Cured PA to stroke	0.0011 (± 30%)	Previous research [24, 43]
PA to death	0.0042 (± 30%)	Previous research [24, 42]
PA to HF	0.0563 (± 30%)	Previous research [36]
PA to stroke	0.129 (± 30%)	Previous research [24, 35, 44]
EHT to death	0.0042 (± 30%)	Previous research [24, 42]
EHT to HF	0.0266 (± 30%)	Previous research [36]
EHT to stroke	0.034 (± 30%)	Previous research [24, 35]
HF to death	0.0154 (± 30%)	Previous research [36]
Stroke to death	0.00505 (± 30%)	Previous research [24, 42]
Cycles (expected life-years)	(years)	
Male	32 (0–50)	Previous research [1]
Female	38 (0–50)	Previous research

*ARR* aldosterone/renin ratio, *CT* computed tomography, *AVS* adrenal venous sampling, *HF* heart failure, *RFA* radiofrequency ablation, *EHT* essential hypertension, *APA* aldosterone-producing adenoma, *PA* primary aldosteronism, *BHA* bilateral adrenal hyperplasia

The rate was used for the deterministic sensitivity analysis

The indications for RFA were examined in multiple clinical departments, namely, radiology, endocrinology, and urology, considering the safety of treatment on the basis of the anatomical relationship between the adrenal gland and surrounding critical organs. In the decision tree model, the selection of surgery or RFA in the comprehensive strategy group was regarded as a chance node.

The clinical outcomes of surgery and RFA were defined on the basis of the primary aldosteronism surgical outcome (PASO) criteria [8, 34]. Complete or partial clinical success was defined as the resolution or reduction of hypertension. The persistence of hypertension at the preoperative same level was classified as a lack of clinical success.

### Markov model

After diagnosis and treatment, a Markov model was developed. We focused on stroke and heart failure (HF) as major complications of hypertension, and four health states, namely, hypertension, stroke, HF, and death, were used in the Markov model (Fig. 1). The transition probabilities in the model were defined on the basis of previous research [14, 24, 33]. Because a previous study reported that the incidence of stroke or other cardiovascular events in PA is considered higher than that in EHT, the transition probabilities reflected this finding [35].

However, because epidemiological data on Japanese patients with PA are limited, we defined some of the probabilities on the basis of foreign epidemiological data in consultation with an endocrinologist [25, 36]. One cycle represented 1 year in this model; 32 and 38 years were adopted on the basis of expected life-years in 50-year-old Japanese men and women, respectively. Costs and effectiveness were discounted by 2% annually [31].

### sAVS

Successful sAVS was defined as blood collection from more than two branches of each adrenal vein on both sides [14, 17]. In the analysis, both successful and unsuccessful cases of sAVS and cAVS were incorporated into the model. The cost-effectiveness analysis was performed by setting the proportion of surgical PA in successful AVS cases to 48% and 46.1% for sAVS and cAVS, respectively, and the postoperative remission rates to 84% and 80.2% for sAVS and cAVS, respectively, considering the lower diagnostic accuracy of cAVS than sAVS on the basis of our hospital data and a previous study [14].

### Costs

The analysis was performed from a healthcare payer perspective. The cost data for the blood testing, CT, and AVS were estimated from the Japan Ministry of Health, Labor and Welfare medical fee schedule. The annual drug costs were calculated from our hospital data. The costs of any screenings, surgeries, complications, and medications were included in the analysis, but non-healthcare-related costs were not included. Under the Japanese health insurance system, AVS procedure fees are reimbursed, so procedure fees were included in the cost from the healthcare payer perspective. On the other hand, the costs of devices such as catheters, microcatheters, and RFA needles were not included from the healthcare payer perspective because they are not reimbursed by insurance.

### Quality-adjusted life-years

Treatment effectiveness was measured using QALYs, which was determined by multiplying survival life-years by utility. The QALYs of patients receiving antihypertensive treatment, patients with heart failure, and patients with stroke were estimated on the basis of previous studies through consultation with internal medicine specialists [5, 37] (Table 1).

### Cost-effectiveness analysis

The outcome measures were the expected costs, expected effectiveness, and ICER. The ICER was calculated via the following formula: ICER = incremental costs/incremental QALYs. If one strategy had more QALYs and fewer costs than the other strategy did, it “dominated” the other strategy. If not, an ICER was calculated, and the strategy preference was determined on the basis of a 5,000,000 JPY per QALY societal willingness-to-pay threshold, in accordance with previous studies [38–40].

### Sensitivity analysis

To determine the robustness of the model, we established deterministic sensitivity analyses and created a tornado diagram on the basis of the range of probabilities shown in Table 1. We assigned a deviation of 30% for probabilities and a deviation of 8% for QALYs. The range of the discount rate was determined to be 0–4% on the basis of Japanese guidelines. We assigned a deviation of 20% for the cost parameters. We also performed a one-way sensitivity analysis by changing the number of cycles to determine how many more years of expected life expectancy would exceed the cost-effectiveness threshold.

In addition, a probabilistic sensitivity analysis was conducted with Monte Carlo simulations on the basis of the estimated distributions of each parameter using 10,000 samples. Beta distributions with standardized differences of 10% and 5% were adopted for probabilities and utilities, respectively, whereas gamma distributions with standardized differences of 10% were adopted for costs.

## Results

### Patients for retrospective data collection

A total of 114 patients who underwent sAVS or cAVS were eligible. The mean age was 54.54 ± 12.56 years, and 50 patients (44%) were men (Table 2).

**Table 2:** Patient characteristics

Mean age (years)	54.54 ± 12.56
Sex	
Male	50 (44)
Female	64 (56)
Adrenal venous sampling	
Segmental AVS	112 (98)
Conventional AVS	2 (1.8)
Disease type	
Unilateral APA	47 (41)
Bilateral APA	1 (0.88)
Bilateral adrenal hyperplasia	66 (58)
Unilateral APA	
Surgery	43 (91.5)
RFA	4 (8.5)

Continuous variables are expressed as the means ± standard deviations

Categorical variables are expressed as *n* (%)

*AVS* adrenal venous sampling, *APA* aldosterone-producing adenoma

### Cost-effectiveness analysis

The expected costs were 2,708,321 JPY and 3,091,802 JPY for the comprehensive strategy for men and women, respectively (Table 3). The expected costs were 2,696,803 JPY and 3,091,595 JPY for the medication-only strategy in men and women, respectively. The expected QALYs for men and women were 21.201 years and 23.309 years, respectively, for the comprehensive treatment strategy and 21.144 years and 23.248 years, respectively, for the medication-only strategy. These analyses revealed ICERs of 201,482 JPY per year and 3,399 JPY per year in men and women, respectively (Table 3).

**Table 3:** Cost-effectiveness results

Strategy	Expected costs (JPY)	Incremental costs compared with the medication-only strategy (JPY)	Expected QALYs	Incremental effectiveness compared with the medication-only strategy	Incremental cost-effectiveness ratio compared with the medication-only strategy
Comprehensive strategy (sAVS with RFA)					
Male	2,708,321	11,518	21.201	0.05716	201,482
Female	3,091,802	207	23.309	0.06077	3,399
Medication-only strategy					
Male	2,696,803		21.144		
Female	3,091,595		23.248		
(i) sAVS without RFA					
Male	2,710,187		21.201		
Female	3,093,772		23.309		
(ii) cAVS with RFA					
Male	2,714,046		21.198		
Female	3,098,474		23.306		
(iii) cAVS without RFA					
Male	2,711,855		21.199		
Female	3,095,899		23.306		

*QALYs* quality-adjusted life-years, *sAVS* segmental adrenal venous sampling, *RFA* radiofrequency ablation, *cAVS* conventional adrenal venous sampling

### Sensitivity analysis

**Fig. 2:**
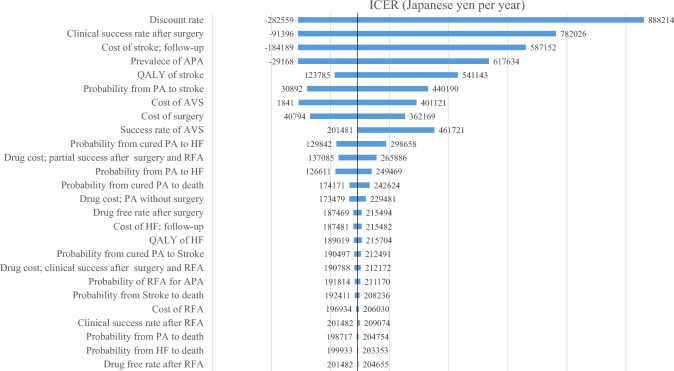
Tornado diagram of the incremental cost‒effectiveness ratios of the comprehensive strategy and the medication-only strategy in 50-year-old men with hypertension. The tornado diagram was constructed on the basis of the estimated ranges of each individual model-input parameter. The upper parameters have a greater effect on the incremental cost-effectiveness ratio. *ICER* incremental cost-effectiveness ratio, *APA* aldosterone-producing adenoma, *PA* primary aldosteronism, *AVS* adrenal venous sampling, *RFA* radiofrequency ablation

The results of the one-way sensitivity analyses are summarized in a tornado diagram (Fig. 2). We identified 26 parameters that were expected to influence cost-effectiveness. The discount rate had the greatest influence on the ICER. The ICER was consistently less than 5 million JPY in this sensitivity analysis.

**Fig. 3:**
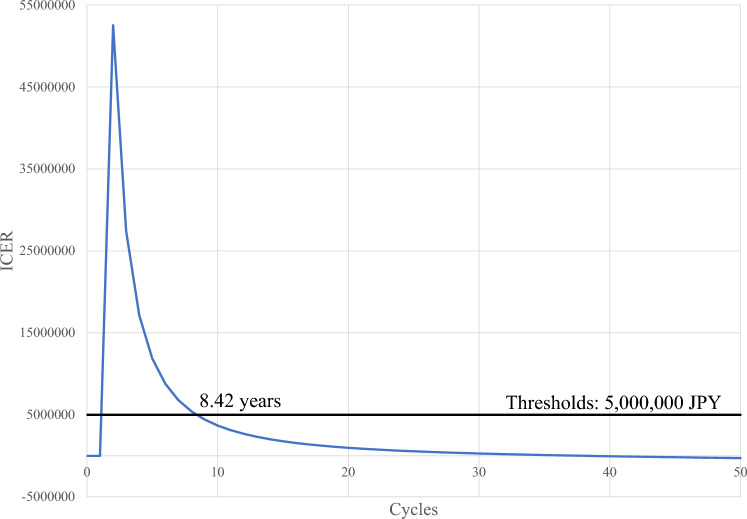
Sensitivity analysis for expected life-years. *ICER* incremental cost-effectiveness ratio, *JPY* Japanese yen

Figure 3 shows the results of the one-way sensitivity analysis when we varied the number of cycles, indicating that the ICER is lower than the threshold value (5 million JPY) when the life expectancy is 8.42 years or longer.

**Fig. 4:**
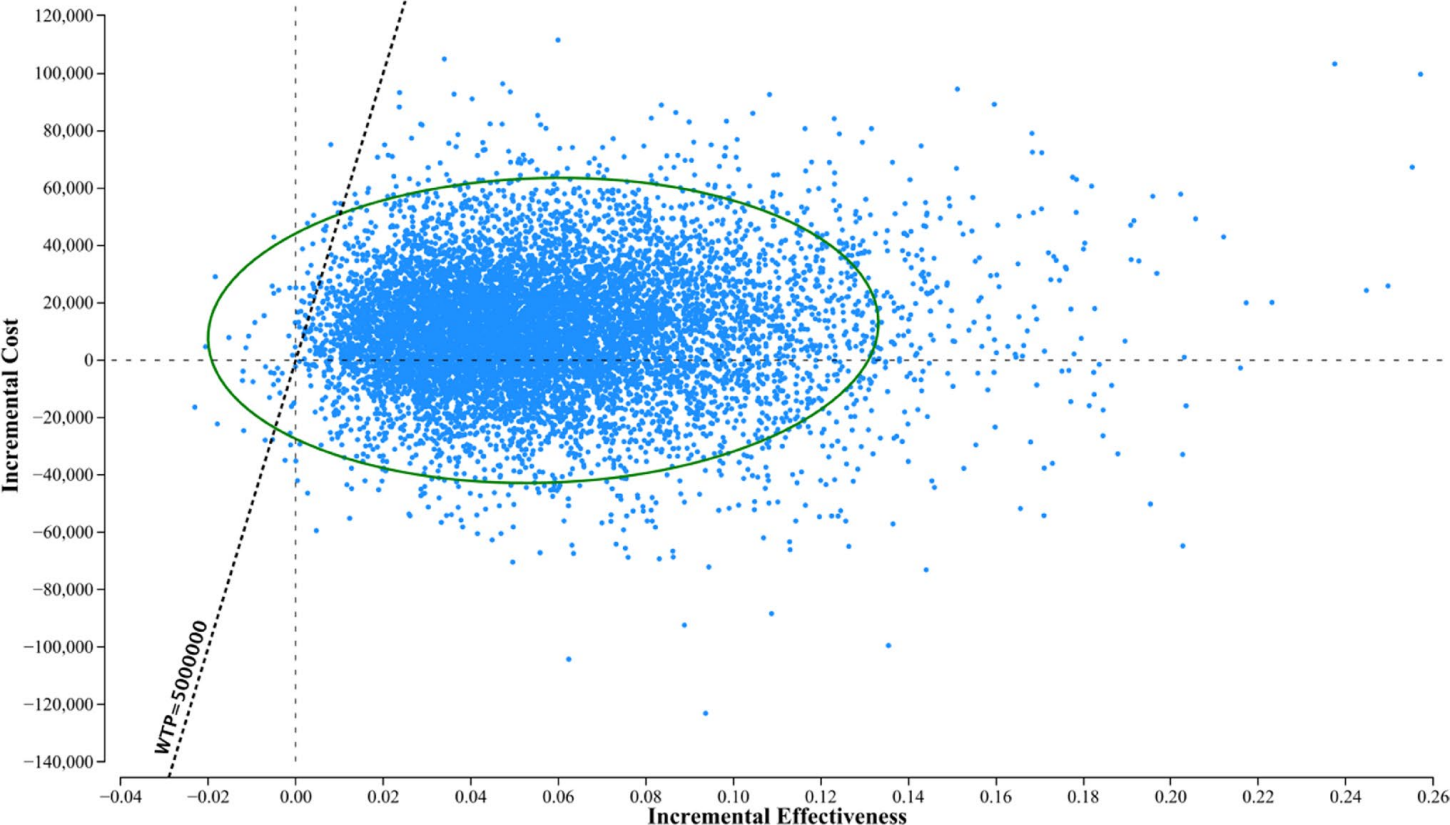
Probabilistic sensitivity analysis for the cost-effectiveness of the comprehensive strategy and medication-only strategy. The dots denote the results of the Monte Carlo simulations of 10,000 samples. More than 80% of the dots are located below the benchmark line for willingness-to-pay (5 million JPY per QALY); that is, the comprehensive strategy is cost-effective compared with the medication-only strategy

More than 80% of the estimates in the probabilistic sensitivity analysis with Monte Carlo simulations were located below the willingness-to-pay of 5 million JPYs/QALY threshold (Fig. 4); that is, the comprehensive strategy was likely to be cost-effective compared with the medication-only strategy, even though the parameters were observed with a range.

### Cost-effectiveness analysis of sAVS and RFA

**Fig. 5:**
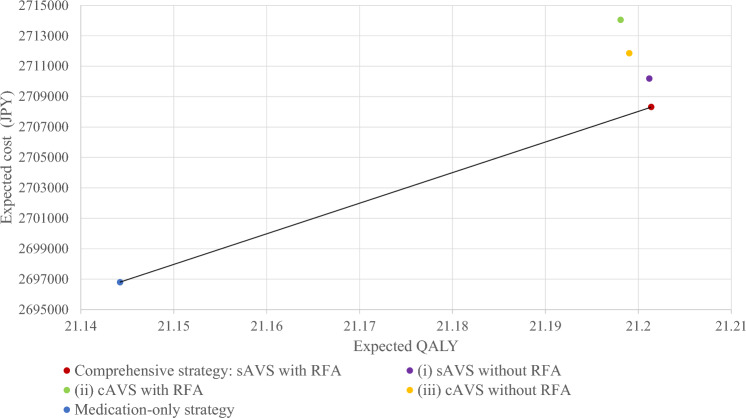
Cost-effectiveness analysis for sAVS and cAVS with/without RFA in 50-year-old men with hypertension. *sAVS* segmental adrenal venous sampling, *RFA* radiofrequency ablation, *cAVS* conventional adrenal venous sampling

A rollback calculation was performed on the estimate point for the expected costs for the expected life-years (Table 3 and Fig. 5) associated with the suboptimal strategies. Compared with the iii) cAVS without RFA strategy, the (i) sAVS without RFA strategy had lower costs and better QALYs. However, the (ii) cAVS with RFA strategy had greater costs and almost equivalent QALYs than did the (iii) cAVS without RFA strategy.

## Discussion

A cost-effectiveness analysis revealed that a comprehensive treatment strategy that includes sAVS and RFA increased the expected costs and QALYs compared with a medication-only strategy, and the resulting ICER was less than 5 million JPY. Deterministic sensitivity analysis and probabilistic sensitivity analysis revealed that the results varied with the input values, but the comprehensive strategy was likely more cost-effective than the medication-only strategy.

Several studies have reported the cost-effectiveness of PA treatment [24–28, 41]. Sato et al. reported that lifetime medical costs and life-years gained were greater for patients with hypertension screened and treated via a comprehensive strategy than for those treated via a standard medication-only strategy. The resultant ICER was under 5 million JPY [24]. Li et al. reported that from the perspective of Chinese healthcare payers, the strategy of screening all hypertensive patients for PA may be more cost-effective than screening only high-risk patients and providing standard antihypertensive treatment for low-risk hypertensive patients [25]. Lubitz et al. compared seven diagnostic strategies for PA in a simulated population of at-risk resistant hypertension patients [26]. The authors reported that, at an accepted willingness-to-pay threshold, screening for PA in the resistant hypertensive population was cost-effective. In addition, CT followed by confirmatory AVS was shown to be the optimal screening strategy for identifying patients with surgically-curable adrenal disease. However, these studies did not include sAVS or RFA, and the cost-effectiveness of the PA treatment strategy on the basis of recent Japanese guidelines remains unclear.

Advances in PA-related interventions, including RFA and sAVS, have expanded the options for PA treatment; however, both sAVS and RFA are costly practices that require multiple specialists to achieve successful outcomes [5, 24]. Notably, this study showed that treatment with RFA based on sAVS provides benefits beyond the payment threshold from an administrative perspective. These findings suggest that accurate lesion localization with sAVS and minimally invasive treatment of APA with RFA may be useful from a health economics perspective. Therefore, widespread use of sAVS for PA and RFA for APA and an increase in the number of patients who are cured of PA will not only have positive outcomes for patients who receive treatment but also have favorable implications for healthcare payers.

In the suboptimal strategy analysis, (iii) cAVS without RFA, which is currently implemented at most institutions in Japan, showed lower QALYs and higher costs than the comprehensive strategy. Compared with this strategy, (i) sAVS without RFA resulted in better QALYs and lower costs. These findings suggest that accurate lesion localization with sAVS contributes to increased cost-effectiveness.

In this analysis, the increased human resources required for sAVS compared with cAVS were not considered. Because this analysis was performed from the healthcare payer perspective, the time cost of patients’ access to specialized hospitals was not considered. At the time of data collection in this study, reimbursement prices for sAVS and cAVS were the same in Japan. If sAVS were to have an independent reimbursement price in Japan in the future, the results of the analysis may be different.

We showed that the ICER was below the threshold value of 5 million JPY when the life expectancy was ≥ 8.42, suggesting that the comprehensive strategy is highly cost-effective for men aged < 81 years and women aged < 85 years in Japan [42]. These ages are greater than the average age of the patients analyzed in this study, which suggests that there are older patients who are potentially indicated for a comprehensive strategy for PA from a health economics perspective.

Our study has several limitations. First, some of the input data used in the analysis were surrogate data from previous studies [24, 37]. The origin of the QALY weight was not based on the data that measured quality of life for the actual conditions. The mortality and QALYs unique to PA patients have not been fully examined in previous studies. In addition, although some RFA complications are fatal, the cost of complications and their impact on QALYs were not included in this Markov model; further accumulation of epidemiological data is needed. Second, we collected some of the input data from our hospital, but the sample size might be considered too small to identify statistical relationships, and the data were collected from a single center. In addition, the cutoff values of laboratory data for diagnosing PA vary worldwide [4]. To determine the robustness of the model, we conducted deterministic and probability sensitivity analyses, which were performed over a wide range of inputs, although the data used for the sensitivity analysis were based on limited clinical data and relevant previous studies. Further data accumulation and econometrically refined analyses are desirable to improve the credibility of the analysis. Third, we considered only stroke and heart failure as complications of hypertension because limited cost data were available. Other complications need to be included in future studies. Fourth, non-healthcare-related costs were not included in the analysis. Even if the cost-effectiveness of treatment is high, if the human resources and time costs of medical practitioners exceed this value, it can be concluded that it represents a loss for society. Because the data concerning non-healthcare-related costs are limited, further data accumulation is desirable to improve the credibility of the analysis. Fifth, the differences in the cost and QALYs between the strategies were slight. In particular, the observed difference in QALYs between the comprehensive strategy and the medication-only strategy was indeed modest, although this small difference in QALYs suggested a benefit of the comprehensive strategy. The small differences in costs and QALYs between strategies may raise concerns about the reproducibility of the results. This aspect requires further research and analysis. To ensure the reproducibility of the results and the validity of the conclusions, we believe that the following points are crucial: 1) conducting larger-scale and longer-term studies, 2) expanding the sensitivity analyses for the suboptimal strategies, and 3) improving and standardizing the QALY measurement methods. These efforts would allow for a more accurate evaluation of the differences between strategies and enhance the reliability of the results. Finally, we considered the ICER of under 5 million JPY to be cost-effective. The introduction of a new treatment strategy would normally incur initial costs. When assessing the cost-effectiveness of a strategy, it is useful to have a national evidence-based threshold to inform the allocation of resources. However, Japan does not have a formal national threshold for the ICER; therefore, we substituted payment thresholds from previous studies [38–40].

## Conclusion

Our cost-effectiveness analysis of diagnosing and treating PA revealed that lifetime medical costs were higher and that QALYs were greater for patients with hypertension screened and treated in accordance with the comprehensive strategy than for those screened and treated with the medication-only strategy, and the resulting ICER was below the social willingness-to-pay threshold of 5 million JPY per QALY.

## Ethical approval

In this retrospective study, our hospital data comprised data for patients treated in our institution, only. The institutional review board approved this study and waived the requirement to obtain informed consent (approval number; 2021–1-979).

## Consent to participate

Informed consent was obtained from participants in the form of an opt-out on the website.

## Consent to publish

The participants consented to the submission of this manuscript to the journal.
